# Comparing Clinical Treatments and Outcomes of Patients with Atrial Fibrillation and Obstructive Sleep Apnea

**DOI:** 10.51894/001c.156350

**Published:** 2026-02-09

**Authors:** Shon Thomas, Kushagra Sharma, Nick Hadji, Monique Luna, Freddie Hildreth, Michael Burton

**Affiliations:** 1 Internal Medicine GME Corewell Health South, Saint Joseph, MI

**Keywords:** Atrial fibrillation, Obstructive sleep apnea, Clinical outcomes, Rate control, Rhythm control

## Abstract

**INTRODUCTION:**

Atrial fibrillation (AF) is a common arrhythmia that is bidirectionally associated with obstructive sleep apnea (OSA), where OSA increases the risk of AF onset and recurrence, while AF may exacerbate OSA symptoms through hemodynamic and autonomic mechanisms. Both conditions pose significant global public health concerns and increase cardiovascular risk. However, the effect of OSA on AF management strategy and outcome has not been explored previously. The purpose of our study was to evaluate clinical outcomes in patients with AF and OSA.

**METHODS:**

Our study included 130 patients over the age of 18 with AF, of which 100 patients were diagnosed with OSA. We evaluated multiple clinical outcomes including comorbidities, treatment strategies, vitals, end organ function, and mortality from June, 2021 to November, 2023 through retrospective chart review. Data was analyzed using chi-square, fisher’s exact test and logistic regression.

**RESULTS:**

Results were significant for a predominance in rate control management strategy (12% rhythm control vs 75% rate control) used in AF with OSA and predominance in rhythm control management strategy (88.9% rhythm control vs 3.7% rate control) used in AF without OSA (p=0.0279). Evaluating mortality in patients with both AF and OSA, COPD (54.6% mortality) and CHF (78.8%) were associated with significantly higher mortality (p<0.0001). There was no significant mortality benefit between rate versus rhythm strategy overall in any patient with AF (OR 0.49, CI 0.153-1.570, p-value =0.229).

**CONCLUSIONS:**

While rate and rhythm control strategies are used interchangeably in managing AF, our study emphasizes a role of OSA in determining rate versus rhythm management strategy for AF treatment. Thus, the presence or absence of underlying OSA may be used as a clinical decision-making factor in managing AF.

## INTRODUCTION

Atrial fibrillation (AF) is the most common sustained arrhythmia. Prevalence of AF is approximately 3 million adults and estimated to be 12-15 million by 2050 in the US.^1^ Obstructive sleep apnea (OSA) is the most common form of sleep related breathing disorder, affecting approximately 10-15% of the general population.^2,3^ Both are global public health concerns. AF and OSA are bidirectionally associated and are common cardiovascular risk factors for stroke.^4^

OSA is defined as Apnea-Hypopnea Index (AHI) greater than or equal to 5 episodes per hour of sleep, measured using overnight polysomnography or home sleep apnea testing. There is increased prevalence of AF with OSA versus without OSA which is independent of age, sex, BMI, and cardiovascular diseases.^5^

OSA and AF share multiple risk factors. Obesity seems to be the metabolic disease that strongly links OSA to AF. With nearly 35% of adult Americans being obese, it is estimated that both OSA and AF will increase exponentially in its prevalence.^2,3,6^ In addition, risk factors such as old age, male gender, alcohol consumption, cigarette smoking, hypertension, and heart failure lead to AF and OSA.^7–9^

The landmark Framingham Heart Study showed that the mortality rates were 50-90% higher in patients with AF compared with those without AF diagnosis.^10^ OSA treatment was associated with improved cardiovascular disease related mortality, but not cardiovascular disease outcomes.^11^ However, the effect of OSA on AF management strategy and outcome has not been explored previously.

The aim of the current study is to evaluate cardiovascular clinical outcomes of AF patients with and without OSA. Specifically, with emphasis on mortality, treatment strategy, outcome, and comorbidities.

## METHODS

This was a retrospective single center study conducted in a community hospital setting including 130 patients with AF from June 2021 to November 2023.

Inclusion criteria included age over 18, OSA confirmed by documented sleep study (i.e. AHI greater than or equal to 5), and AF confirmed by ekg or cardiac telemetry. Exclusion criteria included anyone below age 18, OSA diagnosis without sleep study confirmation and AF diagnosis without EKG confirmation.

We collected three demographic variables (age, race, gender), 16 comorbidity variables (obstructive sleep apnea, type of atrial fibrillation (paroxysmal, permanent, persistent, chronic or other), hypertension, diabetes, chronic obstructive pulmonary disease (COPD), congestive heart failure (CHF), coronary artery disease (CAD), chronic kidney disease (CKD), end stage renal disease (ESRD), hyperlipidemia, stroke, history of myocardial infarction (MI), history of coronary artery bypass graft (CABG), history of percutaneous coronary intervention (PCI), valve disease, history of valve repair), six vital variables (heart rate (HR), systolic blood pressure (SBP), diastolic blood pressure (DBP), height, weight, and BMI), four AF treatment strategy variables (management strategy, history of cardioversion, catheter ablation of AF, and AV node his bundle ablation), and two outcome variables (mortality and left ventricular ejection fraction (LVEF)).

### Primary and Secondary Outomes

The primary outcome looked at the effect of obstructive sleep apnea on clinical outcomes in AF patients. Comparing AF patients with and without OSA, clinical outcomes including mortality, management strategy (rhythm control vs rate control vs catheter ablation) and cardiac function (defined as variable LVEF: below 50% versus 50% and above) were analyzed.

Rate control is defined as pharmacological intervention such as beta blockers, non-dihydropyridine calcium channel blockers or digoxin. Whereas rhythm control is defined as antiarrhythmic pharmacologics such as amiodarone, sotalol, flecainide, procainamide, propafenone, and others.

The secondary outcomes evaluated the effect of different comorbidities on mortality in patients with both AF and OSA. The different diseases evaluated were hypertension, diabetes, COPD, CHF (with any LVEF), CAD, and CKD (EGFR below 60).

### Statistical methods

Descriptive statistics were created based on demographic variables to describe the characteristics of the sample size that include race, sex, height, weight, BMI, age, mortality, type of AF, and OSA. A Shapiro Wilks test was used to test the data to see if it met the normality assumption. Means and standard deviations were reported if the data was normally distributed, and medians along with the 25^th^ and 75^th^ percentiles were used if the data was not normally distributed.

The primary and secondary outcomes were evaluated using chi-square analysis to compare the effect of AF in patients with and without OSA as well as the effect different comorbidities have on mortality in patients with both OSA and AF . If the expected cell count was less than 5, then a Fisher’s exact test was used for analysis.

The effect of management style, rate or rhythm, was explored by using a logistic regession model to predict the odds of mortality given the type of management style the patient was given. The threshold for significance for all statistical testing was 0.05.

## RESULTS

Of the 130 patients (mean (SD) age, 73.52 (9.93) years; 68 male (54%); 59 female (46%)) diagnosed with AF, 100 patients were diagnosed with OSA (Table 1).

**Table 1.** Patient Demographics

**Variable**	**Overall Patients (N=130)**	**OSA (N=100)**	**Non-OSA (N=30)**
	*No. (%) +/- Standard deviation*		
Age	73.52 +/- 9.93	73.44 +/- 9.39	73.81 +/- 11.87
Identified Race White Black or African American Other Missing	104(83) 15(12) 6(5) 5	82(84) 12(12) 4(4) 2	21(78) 3(11) 2(7) 3
Sex Male Female Missing	68(54) 59(46) 3	58(58) 42(42) 0	10(37) 17(63) 3
Type of AF Paroxysmal Permanent Persistent Chronic Other	81(63) 23(18) 20(13) 2(2) 4(3)	64(64) 20(20) 13(13) 1(1) 1(1)	17(57) 3(10) 7(23) 1(3) 2(7)
Hypertension Yes Missing	113(89) 5	89(89) 0	24(89) 3
Diabetes Mellitus Missing	65(51) 3	50(50) 0	12(44) 3
Chronic Obstructive Pulmonary Disease Missing	37(29) 4	28(28) 1	9(33) 3
Congestive Heart Failure Missing	58(46) 3	49(49) 0	9(33) 3
Coronary Artery Disease Missing	59(46) 3	50(50) 0	9(33) 3
Chronic Kidney Disease ^a^ Missing	51(40) 3	40(40) 0	11(41) 3
End State Renal Disease Missing	5(4) 3	5(5) 0	0(0) 3
Hyperlipidemia Missing	94(75) 4	78(79) 1	16(59) 3
Stroke Missing	27(21) 3	20(20) 0	7(26) 3
History of MI Missing	20(16) 3	16(16) 0	4(15) 3
History of CABG Missing	13(10) 3	12(12) 0	1(4) 3
History of PCI Missing	25(20) 3	22(22) 0	3(11) 3
Valve disease Missing	29(23) 3	26(26) 0	3(11) 3
History of valve repair Missing	9(7) 3	8(8) 0	1(4) 3
	*Median (25^th^, 75^th^)*		
Height(cm)	170.2(159, 180.3)	172.7(161.3, 180.67)	162.6(157.5, 170.20)
Weight(kg)	102.5(85, 121)	103.93(90.91, 125.85)	92.13(75.60, 105.87)
BMI	34.54(29.69, 40.4)	34.63(29.84, 40.32)	33.83(27.15, 41.36)
	*Mean +/- Standard Deviation*		
Heart rate Missing	73.29 +/- 14.98 4	72.22 +/- 14.33 0	77.38 +/- 16.93 3
SBP Missing	129.92 +/- 21.19 3	130.49 +/- 20.83 0	127.81 +/- 22.77 3
DBP Missing	73.56 +/- 13.50 3	73.65 +/- 13.69 0	73.22 +/- 13.00 3

Abbreviations: OSA, obstructive sleep apnea; AF, atrial fibrillation; MI, myocardial infarction; CABG, coronary artery bypass graft; PCI, percutaneous coronary intervention; SBP, systolic blood pressure; DBP, diastolic blood pressure.

a: CKD is defined as CKD stage 2 or worse (i.e. EGFR below 60)

Majority were overweight elderly white males with multiple medical comobordities. Comparing OSA and non-OSA patients wth AF, OSA patients had more diabetes (50% versus 44%), CHF (49% versus 33%), CAD (50% versus 33%), ESRD (5% versus 0%), hyperlipidemia (79% versus 59%), valve disease (26% versus 11%), CABG (12% versus 4%), PCI (22% versus 11%) and valve repair (8% versus 4%). While non-OSA patients had more COPD (33% versus 28%) and stroke (26% versus 20%). Vitals were well matched between the two groups. Patients with missing data in the electronic medical record were listed separately.

There was no significant difference in mortality between AF patients that were diagnosed with OSA (33, 33%) and non-OSA patients (12, 40%) (p value = 0.480) (Table 2).

**Table 2.** The Effect of Obstructive Sleep Apnea (OSA) on Clinical Outcomes in AF Patients

**Variable** *No. (%)*	**OSA (N=100)**	**Non-OSA (N=30)**	**P-value**
Mortality	33(33)	12(40)	0.4797
Management Strategy Rhythm Rate Control None Missing	12(12) 75(75) 13(13)	24(89) 1(4) 2(7) 3	0.0279^a^
Catheter Ablation Missing	24(24)	20(74) 3	0.8362
AV Node of His Bundle Ablation Missing	91(94) 3	25(96) 4	1.00
LVEF over 50% Missing	14(16)	5(21) 6	0.5449^a^

Abbreviations: OSA, obstructive sleep apnea; AV, atrioventricular; LVEF, left ventricular ejection fraction

a: Fisher’s exact test used instead of chi-square due to small cell count

Also, there was no significant difference in incidence of LVEF > 50% (p = 0.545). Interestingly, there was a significant difference in the management strategies between OSA and non-OSA patients (p = 0.0279). Non-OSA patients were managed with rhythm control strategy compared with OSA (24 (89%) versus 12 (12%) patients). 75 (75%) OSA patients were managed with rate control while only one (4%) non-OSA patient underwent rate control (Table 2).

Mortality was used as an outcome variable to better characterize the associations between AF, OSA and other comorbidities. While mortality was not significantly elevated in OSA patients, we wanted to further elucidate the relationship between comorbdity and mortality in patients with both AF and OSA. Evaluating mortality in patients with both AF and OSA, COPD patients had a significant increase in mortality (18, 55%) (p < 0.001). Mortality was significantly elevated in CHF patients with both AF and OSA as well (26, 79%) (p < 0.001). There were no significant differences in mortality among comorbidities (Table 3).

**Table 3.** Effect of Comorbidities on Mortality in Patients with AF and OSA

**Variable** *No. (%)*	**No Mortality(N=67)**	**Mortality (N=33)**	**P-value**
Hypertension	58(87)	31(94)	0.3302^a^
Diabetes Mellitus	29(43)	21(64)	0.0556
COPD	10(15)	18(55)	<0.0001
CHF	23(34)	26(79)	<0.0001
CAD	30(45)	20(61)	0.1366
CKD	25(37)	15(45)	0.4346

Abbreviations: COPD, chronic obstructive pulmonary disease; CHF, congestive heart failure; CAD, coronary artery disease; CKD, chronic kidney disease

a: Fisher’s exact test used instead of chi-square due to small cell count

To see if management strategies affected mortality, we performed regression analysis. Patients with rate control management strategy had lower, although insignificant, odds of mortality compared with rhythm control management strategy (OR 0.49, CI 0.153-1.570, p-value =0.229) (Table 4).

**Table 4.** Odds Ratio for Management Style (Rate or Rhythm) and Mortality

**Variable**	**Odds Ratio (95% Confidence Interval)**	**p-value**
Management Style (Rate vs Rhythm Control)	0.490 (0.153, 1.570)	0.2298

## DISCUSSION

Prior studies show OSA is known to increase the risk for AF. OSA was associated with a 1.3-fold increase in the risk of AF for every 1-point increase in apnea-hypopnea index in a retrospective cohort study.^12^ In the Sleep Heart Health Study, a cross-sectional study, OSA reportedly increased AF risk fourfold.^13^ However, no prior study has examined the clinical outcomes of AF based on OSA diagnosis.

Our study identifies a novel management strategy for AF patients based on their underlying diagnosis of OSA. The primary outcome is that patients with AF and OSA were more frequently managed with a rate control strategy since there is significant difference in management strategy between OSA and non-OSA patients (Table 2). Likewise, AF patients without OSA may be appropriately treated with rhythm control strategy. There was no significant difference in cardiac function (LVEF) between the two groups. Overall mortality was not significantly different between OSA and non-OSA AF patients. Moreover, rate control medications such as beta blockers (e.g. metoprolol, bisoprolol, carvedilol) showed no mortality benefit over rhythm control (e.g. amiodarone, dofetilide) Table 4).

Whether a patient is appropriately treated with rate or rhythm control strategy for AF is a controversial topic. The AFFIRM (Atrial Fibrillation Follow-up Investigation of Rhythm Management) trial showed no mortality benefit between rhythm versus rate control treatment strategy at median follow up of 3.5 years.^14^ Thus, rhythm strategy was avoided by most clinicians due to the signficant side effects including proarrhythmia. Prior studies have demonstrated higher AF recurrence following rhythm control strategies, including cardioversion and catheter ablation, in patients with untreated OSA compared with those without OSA. On the other hand, the EAST-AFNET 4 (Early Rhythm-Control Therapy in Patients with Atrial Fibrillation) trial suggested rhythm control was superior to rate control in reducing mortality and cardiovascular outcomes at median follow up of 5.1 years.^15^ However, the above studies have not evaluated the role of OSA diagnosis on AF treatment management strategy.

The current study shows that AF with OSA is predominantly treated with rate control strategy and AF without OSA is predominantly treated with rhythm control strategy. Although, rate control treatment strategy does not decrease odds of mortality, in comparison to rhythm control strategy, in any patient with AF regardless of OSA diagnosis (Table 4). Our study emphasizes a role of OSA in determining rate versus rhythm management strategy for AF treatment. Rate control (75%) being used predominantly in the AF with OSA group and rhythm control (88.9%) being used in AF without OSA group (Table 3). This significant association may help clinicians decide between appropriate management strategy for AF based on underlying OSA diagnosis. While rate and rhythm strategies are both commonly used in clinical settings, sometimes adjunctively, our study identifies the presence or absence of underlying OSA as a clinical decision-making factor in managing AF. This needs to be further explored in a greater clinical context.

The secondary outcome is that cardiovascular comorbidities such as CHF (78.8 %) and COPD (54.6 %) showed significant mortality association in patient with AF and OSA (Table 4). It is unclear whether COPD and CHF increase mortality due to these patients having a worse baseline health status or a combination of untreated OSA and/or AF. Our results align with the current literature findings that AF and OSA is associated with cardiovascular diseases. AF confers a three- to five-fold increase in the risk of stroke, MI and CHF.^16–18^ In the Wisconsin Sleep Cohort, among 1,522 patients followed for 18 years, patients with untreated OSA had five times the risk of CV events when compared with those without OSA.^19^ OSA is associated with increased risk of stroke, hypertension, left ventricular hypertrophy and early atherosclerosis.^20–22^ Contrary to expectations from prior studies, our study population found OSA patients had less stroke than non-OSA patients (20% versus 26%) (Table 1). The incidence of hypertension and MI were relatively matched between the two groups.

Either through synergistic or independent pathophysiological pathways, these diseases remain interconnected. Episodic hypoxemia because of OSA has been identified as potential pathophysiology for these disease correlations. Nocturnal hypoxemia may cause sympathetic nervous system activation, activating the renin-angiotensin system and resulting in elevated BP. OSA is also said to contribute to oxidative stress, hypercoagulability, and inflammation by increasing reactive oxygen species generated secondary to hypoxic episodes.^11^

### Limitations

The study has several limitations. A small sample size could have decreased the power of the study, questioning the true effect of OSA on clinical outcomes. The study being a single center community hospital based retrospective cohort trial limits us from making definitive conclusions between rate versus rhythm control strategies for optimal management of AF with OSA.

OSA patients treated with Continuous Positive Airway Pressure (CPAP) had lower rate of annual AF recurrence compared to untreated patients.^23^ Since we couldn’t measure CPAP compliance in patients in our retrospective study, the effect of CPAP treatment on clinical outcomes was not evaluated. Prospective trials with emphasis on CPAP usage could be an area of focus for future studies.

## CONCLUSIONS

Patients with AF and OSA showed difference in rate control AF management strategy while rhythm control was predominant in patients without OSA diagnosis. The clinical implications of this finding need further exploration. There was no increase in odds of mortality between the rate and rhythm management strategies. Overall mortality was not significantly different between OSA and non-OSA AF patients; however, COPD and CHF may increase mortality in patients with AF and OSA. Thus, patients diagnosed with OSA and AF may warrant further surveillance, in particular, if they have COPD or CHF. Further studies are needed to examine this relationship.

### Disclosures

none
